# Paper-and-Pencil vs. Electronic Patient Records: Analyzing Time Efficiency, Personnel Requirements, and Usability Impacts on Healthcare Administration

**DOI:** 10.3390/jcm13206214

**Published:** 2024-10-18

**Authors:** Matthias Fabian Berger, Johanna Petritsch, Andrzej Hecker, Sabrina Pustak, Birgit Michelitsch, Chiara Banfi, Lars-Peter Kamolz, David Benjamin Lumenta

**Affiliations:** 1 Research Unit for Digital Surgery, Division of Plastic, Aesthetic and Reconstructive Surgery, Department of Surgery, Medical University of Graz, 8010 Graz, Austria; matthias.fabianberger@gmail.com (M.F.B.); johanna.petritsch@stud.medunigraz.at (J.P.); andrzej.hecker@medunigraz.at (A.H.); sabrina.pustak@medunigraz.at (S.P.); birgit.michelitsch@medunigraz.at (B.M.); lars.kamolz@medunigraz.at (L.-P.K.); 2 Statistical Institute, Medical University of Graz, 8010 Graz, Austria; chiara.banfi@medunigraz.at

**Keywords:** digital medicine, electronic patient record, electronic health record, clinical decision support, data science

## Abstract

**Background:** This study investigates the impact of transitioning from paper and pencil (P&P) methods to electronic patient records (EPR) on workflow and usability in surgical ward rounds. **Methods:** Surgical ward rounds were audited by two independent observers to evaluate the effects of transitioning from P&P to EPR. Key observations included the number of medical personnel and five critical workflow aspects before and after EPR implementation. Additionally, usability was assessed using the System Usability Scale (SUS) and the Post-Study System Usability Questionnaire (PSSUQ). **Results:** A total of 192 P&P and 160 EPR observations were analyzed. Physicians experienced increased administrative workload with EPR, while nurses adapted more easily. Ward teams typically consisted of two physicians and three or four nurses. Usability scores rated the system as “Not Acceptable” across all professional groups. **Conclusions:** The EPR system introduced usability challenges, particularly for physicians, despite potential benefits like improved data access. Usability flaws hindered system acceptance, highlighting the need for better workflow integration. Addressing these issues could improve efficiency and reduce administrative strain. As artificial intelligence becomes more integrated into clinical practice, healthcare professionals must critically assess AI-driven tools to ensure safe and effective patient care.

## 1. Introduction

Electronic patient records (EPRs), also called electronic health records (EHRs), have become increasingly prevalent in the healthcare industry, revolutionizing the way patient information is recorded and stored. This shift from traditional paper and pencil (P&P) records to electronic health records has brought about numerous changes and advancements in healthcare documentation methods.

EPRs offer transformative advantages, playing an instrumental role in reshaping global healthcare environments. One central advantage is the creation of standardized and structured patient information databases, facilitating more efficient information retrieval and supporting robust clinical decision-making processes [1]. Complementing on the recognized improvement in patient care efficiency, EPRs also enhance diagnostic accuracy and public health outcomes by enabling timely, precise access to patient information, as well as seamless data exchange and extraction between healthcare providers. They also empower patients by providing control over their health data, ensuring transparency and clarity in its usage [2,3]. Within the healthcare sector, the significance of information technology (IT) in EPRs has been emphasized, notably in offering comprehensive information crucial for delivering high-quality care [4,5]. On a broader scale, EPRs aim to support nationwide health infrastructures by ensuring system interoperability, and establishing a unified framework for addressing and understanding global health issues [6]. From a historical perspective, the emergence and evolution of patient management systems in response to growing data volumes and technological advancements suggest a trajectory where EPRs become integral to healthcare infrastructures [7]. Together, these perspectives highlight the pivotal role of EPRs in promoting more informed, efficient, and adaptable healthcare systems.

Electronic patient records, on the other hand, have increasingly come under scrutiny for their apparent misalignment with clinical workflows, and they have been identified as a salient factor contributing to physician burnout [8]. Clinicians often encounter difficulties in distinguishing between analogous patient data due to suboptimal interface designs. This results in disruptions to the workflow and cognitive overburdening as they grapple with vast volumes of data [9]. This trajectory in EPR development is not solely a reflection on senior clinicians, many of whom delegate their administrative burdens to residents (interns/junior medical staff) or, if available, to physician assistants (=PAs, who are less common or not available in public hospitals in Europe). This is a concern directly reverted to EPR developers, who, being predominantly fixed on function-based and list-centric designs, tend to be blinded or informed about the intricacies of clinical workflows [10]. From the perspective of a majority of healthcare providers, there is usually no clinician engagement during the software development process. When involvement does occur, it frequently arises during the implementation phase, a later stage that requires significant time and elicits limited interest from clinicians outside their primary area of expertise [11].

This study aimed to evaluate the effects of transitioning from a P&P to an EPR system for medication and documentation during ward rounds in a major hospital’s surgical division in a public national European Union healthcare system (Austria), where PAs are usually not available and the documentation requires to be performed by medical professionals. Specifically, the research focused on how this transition influenced the ward-based workflow (i.e., impact of administrative workload and personnel requirements) for clinical staff using planned two-week observational audits during weekday patient rounds in a surgical division.

## 2. Methods

This prospective observational study was approved by the institutional ethical board of the Medical University of Graz (vote #: 34-208 ex21/22). It was conducted at the Division of Plastic, Aesthetic and Reconstructive Surgery, Department of Surgery, at the Medical University of Graz between November 2021 and May 2022.

**Figure 1:**
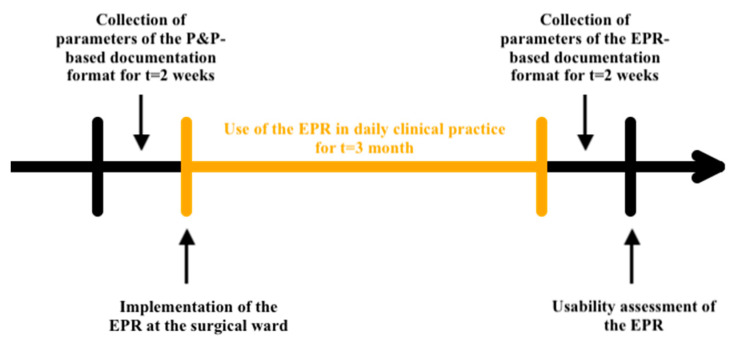
Clinical workflow assessment.

We investigated to what extent the EPR use affected the time clinical personnel spend on hands-on patient care during surgical ward rounds and adopted two distinct workflows (Figure 1) to evaluate clinical routines before and after the introduction of the EPR system.

### 2.1. Workflow A: Observational Audit During Clinical Ward Rounds

Throughout the course of routine clinical ward rounds, two independent observers diligently executed two distinct, continuous audits, each extending over a period of two work weeks. Observations started at the patient’s initial contact on the ward (=first visit) and concluded on the day of discharge (=whole sample). There were no restrictions on the number of observations per patient throughout multiple days of their stay. The initial audit was conducted during a phase where paper and pen (P&P) constituted the dominant method of documentation. This was contrasted with a subsequent audit, undertaken three months following the transition to the electronic patient record (EPR) system. The system utilized in this study was the OpenMEDOCS hospital information system. The OpenMEDOCS System is based on SAP’s ERP (electronic resource planning) suite, and was not developed specifically for patient care management, but has been adopted to account for it. OpenMEDOCS integrates three key components: IS-H, responsible for managing administrative functions such as patient admission, transfer, and discharge; IS-H*Med, which handles medical documentation; and the SER-Archiv, a digital archive for storing and retrieving patient records. and was developed by KAGes (Steiermaerkische Krankenanstaltengesellschaft). OpenMEDOCS was designed to streamline both clinical and administrative workflows, ensuring comprehensive data management while supporting the integration of medical and administrative functions across healthcare institutions [12]. The total duration of each visit was calculated as the cumulative time (in seconds) encompassing five key components: (1) preparatory time prior to entering the patient’s room; (2) the duration of the physicians’ documentation time in the patient’s room; (3) the time allocated by nurses for documentation in the patient’s room; (4) the extent of direct patient interaction by the physicians (e.g., conducting clinical examinations, addressing patient inquiries, discussing treatment plans, …); and (5) the time invested in dressing changes. Instances where the duration was recorded as 0 s were treated as missing data. The objective was to conduct a comparative analysis of these five parameters between the P&P and EPR documentation modalities. To mitigate baseline disparities arising from variations in total visit duration, proportions were calculated by dividing the time dedicated to each of the five (1–5) aspects by the overall ward round time. Only these proportional values were considered relevant for our analysis, as they provide a more accurate reflection of task allocation within the context of varying total visit times. Furthermore, the number of medical personnel present during the ward round was collected. The data for the paper and pencil entry mode were collated between 30 November 2021 and 10 December 2021, whereas the data pertaining to the electronic mode were gathered between 7 March 2022 and 18 March 2022.

### 2.2. Statistical Analysis for Workflow A

Continuous variables were not normally distributed and were descriptively summarized using median and interquartile range (IQR]. Categorical variables were presented as absolute and relative frequencies. Note that the number of medical personnel was treated as a categorical variable to facilitate comparison between the different modalities. Between-subject comparisons of continuous variables were performed with the Wilcoxon rank sum test. Associations among categorical variables were tested by means of Fisher’s exact test. Visit durations for the same patient on consecutive days might have been influenced by medical staff’s prior familiarity with the patient. To mitigate this potential bias, sensitivity analyses were performed where only the first patient visits were included. *p*-values < 0.05 were deemed statistically significant. The analysis was executed using R software (version 4.2.2) [13].

### 2.3. Workflow B: Post-Implementation EPR Usability Survey

**Figure 2:**
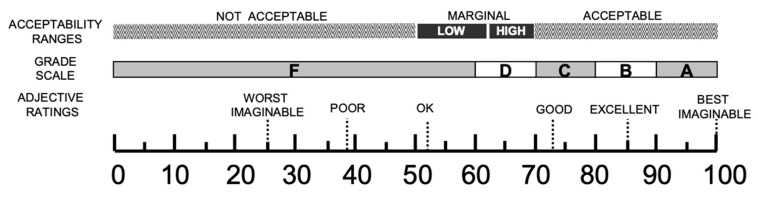
Modified SUS ranking by Bangor et al. [17].

Separately from the observational audits, we polled the clinical staff (nurses, residents, and attendings) three months after the EPR’s introduction. We evaluated the EPR usability as perceived using the firsthand experiences of two established instruments served as poststudy questionnaires: the System Usability Scale (SUS) and the Post-Study System Usability Questionnaire (PSSUQ) [14]. The SUS is widely used and accounts for 43% of post-study questionnaires in the literature [15]. The 10-question questionnaire is based on a 5-point Likert scale (1 = strongly disagree; 5 = strongly agree). The calculated combination yields an overall usability score ranging from 0–100% [16]. A modified score ranking by Bangor et al. (Figure 2) was used to interpret the overall usefulness of each patient [17]. The PSSUQ-3 is conceptualized to evaluate users’ subjective satisfaction with computerized systems and applications. The 16-question questionnaire is based on a 7-point Likert scale (1 = strongly agree; 7 = strongly disagree). The score is divided into an overall average (derived from the mean of all 16 components). This encompasses a “system usefulness” subscale, gauging the system’s intuitiveness and learnability (average of items 1–6); an “information quality” subscale, reflecting the feedback relayed to the user by the system (average of items 7–12); and an “interface quality” subscale, evaluating the use’s affinity for the system and its alignment with anticipated functionalities (average of items 13–16). Normally, a lower score on the PSSUQ correlates with a higher perceived usability of the system [18]. For the purpose of enhanced readability, the PSSUQ scores have been inverted so that an increased score is directly associated with improved system usability. Both questionnaires, the SUS and PSSUQ, are available in the Supplementary Materials (Tables S1 and S2).

### 2.4. Statistical Analysis for Workflow B

The statistical evaluation entailed determining the means and standard deviations (SD) for continuous variables, alongside the computation of frequencies and proportional frequencies for categorical variables. In cases where data did not adhere to a normal distribution, the median and the interquartile range (IQR) were employed.

## 3. Results

### 3.1. Duration of Administrative Work on Patient Care During Surgical Ward Rounds

We collected 191 observations utilizing the P&P system and 160 observations employing the EPR system. No significant differences were observed between the P&P system and the EPR system in the overall duration of ward rounds and in three out of five critical components: the proportional preparatory time before entering the patient’s room (*p* ≥ 0.470), the proportional extent of direct patient interaction by the physicians (*p* ≥ 0.575), and the proportional time spent changing dressings (*p* ≥ 0.674). However, significant differences between data entry modalities were observed in the proportion of time spent inside the patient’s room by physicians (*p* < 0.001) and nurses (*p* < 0.001). Physicians spent less time inside the patient’s room using the P&P modality (median = 0.14, IQR = [0.06, 0.24]) as compared to the EPR system (median = 0.19, IQR = [0.12, 0.29]). In contrast, the proportion of time spent by nurses inside the patient’s room was higher with the P&P modality (median = 0.13, IQR = [0.08, 0.18]) as compared to the EPR system (median = 0.10, IQR = [0.06, 0.13]) (Table 1). Note that these significant differences were not replicated in our sensitivity analysis, including only first visits (see Table S3).

### 3.2. Medical Personnel Distribution

**Figure 3:**
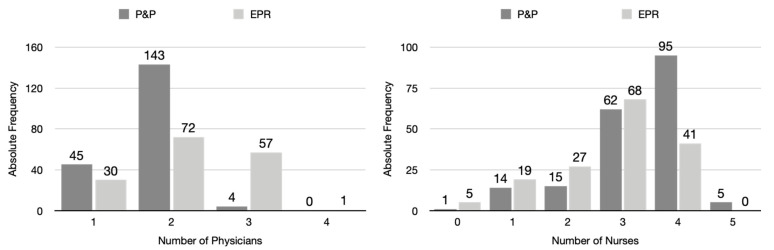
Overall distribution of observed physicians (**left plot**) and nurses (**right plot**).

The proportion of physicians attending to patients differed between the two data entry modalities (Fisher’s exact test, *p* < 0.001). As shown in Figure 3, the presence of two physicians was the most common occurrence in the P&P modality (75%), whereas in the EPR system, two (45%) or three (36%) physicians were most common.

The proportion of nurses attending to patients differed between the two data entry modalities (Fisher’s exact test, *p* = 0.003). In the P&P modality, the presence of four nurses was the most common occurrence (50%), whereas in the EPR system, three nurses (43%) were most common (see Figure 3, right plot).

### 3.3. Usability by Professional Group

Among the participants, the response rate for registered nurses was 53% (9 out of 17), for residents it was 88% (7 out of 8), and for attending physicians it was 50% (7 out of 14).

**Figure 4:**
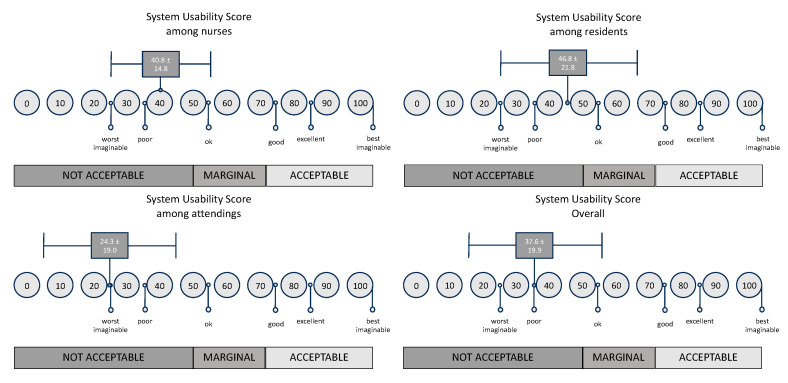
SUS scores across professions.

The System Usability Scale (SUS) (Figure 4) revealed an overall mean score of 37.6 (SD = 19.9). When disaggregated by professional designation, nurses reported an average score of 40.8 (SD = 14.8), residents had a slightly higher mean at 46.8 (SD = 21.8), while attending physicians yielded the lowest mean score of 24.3 (SD = 19.0). In interpreting the findings presented by Bangor et al. [17], it is concluded that all scores are rated as “Not Acceptable”. (The differences were statistically not significant (*p* = 0.085, Kruskal–Wallis).

In the Post-Study System Usability Questionnaire (PSSUQ) (Table 2), the overall mean score was 3.4 (SD = 1.1). Nurses reported a mean score of 3.3 (SD = 1.2), residents had 4.0 (SD = 1.2), and attending physicians averaged at 3.0 (SD = 0.9). Again, the Kruskal–Wallis test rendered a *p*-value of 0.304, indicating no significant differences across the groups.

A deeper examination of the PSSUQ sub-domains further elaborated on these trends. For the SYSUSE segment, the mean score across all respondents was 3.5 (SD = 1.3), with a Kruskal–Wallis *p*-value of 0.243. The INFOQUAL sub-domain resulted in an overall mean of 3.3 (SD = 1.1) and a *p*-value of 0.315. Lastly, for the INTERQUAL sub-domain, participants reported a mean score of 3.4 (SD = 1.3), and the *p*-value stood at 0.717 (see Figure S1 for detailed chart view).

## 4. Discussion

The aim of this study was to investigate the change in administrative workload on the time spent for patient care and the change in the number of personnel requirements following the implementation of an electronic patient record (EPR) system, and to assess the systems’ usability and user acceptance across the involved professional groups.

### 4.1. Administrative Workload Time

The distribution of time during ward rounds has the potential to affect the concentration levels of healthcare professionals and the frequency of medical errors [19]. In utilizing the EPR system, physicians demonstrated a significantly higher workload time on using the EPR than it was by using P&P. From a proportional standpoint, that means that physicians spend more time on documentation than spending time with the patients themselves. Nurses showed the exact opposite in our study. Spending less time on administrative work means more time for addressing the patient from a nursing point of view. With a more detailed examination of ward round duties in both the physician and nursing groups, our results align with a similar study from 2008 found in the literature [20].

In modern healthcare settings, medical professionals are increasingly expected to possess administrative skills in addition to their clinical expertise. A significant amount of a physician’s time is dedicated to administrative tasks, which can detract from direct patient care. This shift towards more administrative responsibilities is linked to lower job satisfaction among doctors [21]. The adoption of the EPR has been identified as a factor that contributes to this increased administrative load. Ammenwerth and Spötl’s study shows that medical professionals spend almost as much time on documentation as they do on taking care of patients directly [22]. Research by Woolhandler and Himmelstein indicates that about one-sixth of a physician’s working hours are spent on administrative work unrelated to patient care [23]. This substantial investment of time in administrative duties can reduce the opportunities for patient interaction, potentially impacting the quality of healthcare services provided. However, while this increased time on documentation with the EPR system may initially seem like an administrative burden, it also reflects the need for training on the system and the standardization of data entry [24,25]. This documentation, though time consuming, serves critical purposes for medico-legal protection, quality metrics, and healthcare reimbursement, which are essential aspects of healthcare [26].

Capturing the spirit of times, the study by Liu et al. [27] demonstrates that the use of an AI-powered clinical documentation tool improved efficiency for many clinicians, reducing time spent on electronic health records and alleviating frustration. Although the benefits and improvements were not universally experienced by all participants, we believe that AI-supported administrative tasks will gain significant momentum in the near future.

### 4.2. Number of Medical Personnel Requirements

Both physicians and nurses demonstrated notable differences in their staffing needs depending on the system used, highlighting that the demand for personnel is dependent on the chosen documentation method. The analysis revealed that a higher number of personnel was required to operate the EPR among physicians compared to P&P. Conversely, nurses demonstrated an opposite trend, whereby fewer nurses were needed when using the EPR as opposed to P&P. Also, the variation in staff composition between the two phases, a result of routine hospital scheduling adjustments beyond the study’s control, reflects the inherent dynamics of real-world clinical settings. As observed in similar studies using snapshot audits, such fluctuations are characteristic of everyday clinical practice and contribute to capturing authentic healthcare conditions [28].

In assessing the number of personnel required, it is crucial to consider not only the workload and care load during the study period but also the minimum personnel requirements dictated by the complexity of patient care. While some administrative tasks can be delegated to support staff such as nurses or physician assistants (PAs), many responsibilities remain non-delegable and must be performed by specialized medical personnel, particularly tasks requiring clinical decision making. This highlights the need for each healthcare professional to compile documentation according to their professional role rather than delegating these tasks entirely.

In terms of workflow innovations, the introduction of PAs into healthcare teams has demonstrated potential for alleviating some of the administrative workload for physicians by delegating routine documentation tasks to trained support staff [29,30]. However, it is important to note that only a few countries have fully integrated PAs into their workforce [31]. Moreover, delegation must be approached carefully, as certain tasks remain the exclusive responsibility of specialized medical personnel and cannot be transferred indiscriminately.

Additionally, any innovation, such as the adoption of EPR systems, should also include a revision of operating models by separating clinical interaction time from documentation tasks, allowing improvement in efficiency without compromising patient care. Another approach is to accept a greater initial time investment in exchange for higher-quality documentation. More thorough and standardized documentation has the potential to save time in subsequent processes, such as data review, transmission, and extraction. This could reduce the need for revisions and accelerate workflow over the long term.

### 4.3. Usability

There is no statistically significant difference in both the System Usability Scale (SUS) and Post-Study System Usability Questionnaire (PSSUQ) scores among the professional groups.

On examining the SUS scores, while the mean scores differed among nurses, residents, and attending physicians, with attending physicians presenting the lowest usability score, these variations were not found to be statistically significant (*p* = 0.085). According to the benchmark scale by Bangor et al. [17], the overall usability yielded an unsatisfactory result. Considering this, it is deemed “Not Acceptable”.

Similar results were found when interpreting the PSSUQ. PSSUQ scores across professional groups indicate room for usability improvements. Nursing staff rated system usability at a mean score of 3.3, while residents had a more favorable 4.0 mean score. Attending physicians were least satisfied, registering a mean score of 3.0 with a low standard deviation, suggesting a homogeneous yet less favorable opinion. The combined mean score for all groups was 3.4, slightly above average but still suggesting the need for enhancements. Further scrutiny into specific categories such as system usability (SYSUSE), information quality (INFOQUAL), and interface quality (INTERQUAL) revealed similar trends. Notably, residents gave the highest scores in the SYSUSE category with a mean of 4.3, whereas attending physicians generally provided lower scores across most categories.

These results align with usability challenges of the EPR system in the literature [32,33,34]. Also, Kaipio et al. mentioned the different usability aspects throughout professional groups, especially physicians and nurses [35]. In our study, common usability issues included slow system responsiveness, data overload, missing or hidden information, poor visibility of required data, and the need for a high number of clicks to complete simple tasks. Although usability issues may vary depending on factors such as EPR brand, level of user training and familiarity, and particular settings in which the system is deployed, these challenges are consistent with findings from the existing literature on EPR usability and underscore areas in need of improvement in system design, with the aim of better supporting clinical workflows [9,36]. Addressing these issues is crucial to prevent disruptions in physician workflow, reduce documentation time, mitigate clinician frustration and burnout, and minimize potential risks to patient safety [37,38,39].

While our study identified significant usability challenges associated with the EPR system, it is important to clarify that our intention is not to position the EPR itself as a fundamental problem. EPR systems are widely acknowledged for their potential to improve patient care by facilitating access to comprehensive patient information, reducing medical errors, and enhancing communication between healthcare professionals [40]. However, in our specific clinical setting, the usability issues encountered, particularly by attending physicians, underscored the need for further refinement and customization of the system to better align with the daily workflows of medical professionals.

The challenges we observed should not be interpreted as inherent flaws of electronic patient records but rather as areas for improvement in system design and implementation. By addressing these usability violations, such systems can better support clinical workflows and reduce the administrative burden on healthcare staff, ultimately enhancing the quality of care provided.

Furthermore, it is essential to recognize that the effectiveness of any EPR system is highly dependent on its integration into the clinical environment and the input of end-users during the design and optimization phases. Our findings suggest that while the EPR system is a critical tool in modern healthcare, its usability—particularly in highly specialized fields like plastic surgery—requires ongoing refinement to fully realize its benefits. This aligns with broader literature emphasizing the importance of user-centered design in healthcare technology development [41,42].

In a hospital system that has not been fully optimized for workflow efficiency, the assignment of purely administrative tasks, such as routine documentation, to highly trained clinical personnel represents a significant economic inefficiency. These tasks, which do not require clinical decision making, could be effectively delegated to other staff, such as PAs. This issue is compounded by the lack of standardized workflows across, and even within, individual hospital systems, leading to habitual inconsistencies that further convolute clinical procedures. Such complexities manifest in various ways, from the excessive number of clicks required to execute specific tasks to the lack of intuitive interface design, necessitating convoluted workarounds. Furthermore, existing enterprise resource planning systems, such as SAP (Systems, Applications, and Products in Data Processing), often do not align seamlessly with the unique demands of clinical workflows. While customization of these platforms is possible, it often incurs substantial follow-up investment costs and typically involves limited input from clinicians. This creates a catch-22 situation, where the very resources needed for optimization are instead contributing to existing inefficiencies. In addition, the potential benefits of economies of scale in the software industry have not been fully utilized in clinical workflows, resulting in missed chances to improve efficiency and cost-effectiveness.

## 5. Limitations

Our observations were confined to a single division and were based on a clinical routine scenario, which demonstrated variability among clinical staff members, with the resident being the only consistent presence. This may have influenced the response rates, as residents’ continuous involvement likely contributed to their higher engagement. In contrast, the variability among other staff members could have contributed to the lower response rates (53% for nurses and 50% for attending physicians). Additionally, the research did not encompass an objective evaluation of in-patient interactions involving varying complexities of patient histories with EPR usability.

## 6. Conclusions

Increased administrative workload appeared to affect only physicians during the transition to the EPR system, whereas nurses did not encounter difficulties switching systems.

However, usability scores indicated that the EPR system falls short of user acceptance expectations, with all professional categories deeming the system “Not Acceptable”. This study indicates that there may be potential advantages to a new electronic system for data entry, but there are explicit usability flaws that prevent its acceptance. Innovations like EPR systems should rethink operational workflows by separating clinical interaction from documentation. Though documentation requires more initial time, it may lead to higher-quality data and fewer revisions later, ultimately saving time in data extraction and transmission. While usability issues with the EPR system were significant, the system itself is not the main problem. EPR systems offer clear benefits, like better access to patient information and reduced medical errors, but these advantages depend on addressing usability issues and customizing the system to fit specific workflows. Our findings highlight that EPR success requires integration into clinical settings and user involvement in design to improve efficiency and reduce administrative strain. Looking forward, as artificial intelligence becomes more integrated into clinical practice, it will be the responsibility of healthcare professionals to critically assess AI-driven workflow, ensuring effective and safe clinical care.

## Figures and Tables

**Table 1:** Comparison of data entry modalities in the whole sample (sensitivity analysis).

	Whole Sample				
	P&P		Electronic		
	N	Median (IQR)	N	Median (IQR)	*p*
Overall ward round time in s *	191	649 (189, 1186)	160	637 (214, 1132)	0.799
Preparatory time before visit in s *	187	42 (20, 84)	147	29 (15, 84)	0.179
Proportional preparatory time before visit	187	0.07 (0.04, 0.20)	147	0.07 (0.02, 0.22)	0.470
Documentation time inside patient room (physicians) in s *	121	76 (45, 164)	133	96 (44, 162)	0.378
Proportional documentation time inside patient room (physicians)	121	0.14 (0.06, 0.24)	133	0.19 (0.12, 0.29)	<0.001
Documentation time inside patient room (nurses) in s *	132	115 (62, 179)	105	83 (55, 120)	0.001
Proportional documentation time inside patient room (nurses)	132	0.13 (0.08, 0.18)	105	0.10 (0.06, 0.13)	<0.001
Interaction time with patients in s *	190	175 (78, 343)	153	176 (91, 328)	0.861
Proportional interaction time with patients	190	0.34 (0.25, 0.46)	153	0.33 (0.27, 0.42)	0.575
Time for dressing changes in s *	114	435 (280, 684)	94	409 (290, 644)	0.676
Proportional time for dressing changes	114	0.44 (0.35, 0.52)	94	0.43 (0.34, 0.49)	0.674

s * = seconds; all *p*-values were calculated using the Wilcoxon rank-sum test.

**Table 2:** PSSUQ scores across professions.

PSSUQ Index *	Nurse Mean, (SD) [%]	Resident Mean, (SD) %	Attending Mean, (SD) %	Overall Mean, (SD) %	*p*-Value
Overall	3.3 (1.2) [38.33]	4.0 (1.2) [50.00]	3.0 (0.9) [33.33]	3.4 (1.1) [40.00]	0.304
System Usability (SYSUSE)	3.3 (1.5) [38.33]	4.3 (1.3) [55.00]	3.0 (0.9) [33.33]	3.5 (1.3) [41.67]	0.243
Quality of the information (INFOQUAL)	3.1 (1.2) [35.00]	3.8 (1.0) [46.67]	3.0 (0.9) [33.33]	3.3 (1.1) [38.33]	0.315
Quality of the interface (INTERQUAL)	3.5 (1.4) [41.67]	3.7 (1.6) [45.00]	3.1 (1.0) [35.00]	3.4 (1.3) [40.00]	0.717

* Rating score from 1 = worst [0%] to 7 = best [100%].

## Data Availability

The data presented in this study are available upon request from the corresponding author.

## Supplementary Materials

The following supporting information can be downloaded at: https://www.mdpi.com/article/10.3390/jcm13206214/s1, Table S1: System Usability Scale (SUS); Table S2: Post-Study System Usability Questionnaire (PSSUQ); Table S3: Comparison of data entry modalities in the subsample of first visits (sensitivity analysis); Figure S1: Detailed Chart View Across Professions for PSSUQ.
