# Supplementary material for: Paper-and-Pencil vs. Electronic Patient Records: Analyzing Time Efficiency, Personnel Requirements, and Usability Impacts on Healthcare Administration
Source: J Clin Med. 2024 Oct 18;13(20):6214. doi: 10.3390/jcm13206214 (PMC11508257; doi:10.3390/jcm13206214)
Supplement: Supplementary file 1 [file jcm-13-06214-s001.zip › jcm-3242939-supplementary.pdf]

Supplementary Material

Table S1: System Usability Scale (SUS)

Nr.	SUS	++	+	0	-	- -
1	I can imagine using the system regularly.					
2	I find the system unnecessarily complex.					
3	I find the system easy to use.					
4	I think I need support from technical personnel to use the system.					
5	I find that the various functions of the system were well integrated.					
6	I find that there are too many inconsistencies in the system.					
7	I can imagine that most people would learn to use the system quickly.					

8	I find the system's operation extremely cumbersome.					
9	I felt very confident using the system.					
10	I had to learn a lot before I could use the system effectively.					

Table S2: Post-Study System Usability Questionnaire (PSSUQ)

Nr.	PSSUQ	+++	++	+	0	-	--	---	n/a
1	Overall, I am satisfied with how easy it is to use this system.								
2	The system’s operation was intuitive.								
3	I was able to complete the tasks quickly using the system.								
4	I found the system’s operation pleasant.								

5	The system was easy to learn.								
6	I believe I became productive quickly using the system.								
7	The system clearly indicated error messages and how to resolve them.								
8	If I made a mistake using the system, I could recover from it easily and quickly.								
9	The information provided by the system (e.g., online help, on-screen messages, documentation) was clear.								
10	It was easy to find the information I needed in the system.								
11	The information provided by the system helped me to complete my tasks.								
12	The layout of the information on the screen was clear.								
13	The system’s interface was intuitive to use.								
14	I liked the system’s interface.								

15	The system has all the functions and capabilities I expected.								
16	I am overall satisfied with the system.								

Table S3: Comparison of data entry modalities in the subsample of first visits (sensitivity analysis)

	**Only first visit**				
	P & P		Electronic		
	N	Median (IQR)	N	Median (IQR)	p
Overall ward round time in s*	62	326 (109, 1200)	52	371 (138, 1163)	0.539
Preparatory time before visit in s*	59	43 (14, 84)	48	30 (17, 74)	0.491
Proportional preparatory time before visit	59	0.10 (0.05, 0.28)	48	0.07 (0.03, 0.28)	0.312
Documentation time inside patient room (physicians) in s*	36	108 (50, 191)	45	77 (41, 142)	0.352
Proportional documentation time inside patient room (physicians)	36	0.16 (0.10, 0.26)	45	0.19 (0.14, 0.29)	0.262
Documentation time inside patient room (nurses) in s*	29	141 (79, 186)	27	86 (33.50, 110)	0.040
Proportional documentation time inside patient room (nurses)	29	0.12 (0.08, 0.18)	27	0.12 (0.07, 0.13)	0.282
Interaction time with patients in s*	62	123 (49, 298)	49	135 (65, 316)	0.320
Proportional interaction time with patients	62	0.41 (0.27, 0.64)	49	0.37 (0.29, 0.46)	0.288
Time for dressing changes in s*	25	585 (371, 809)	25	481 (304, 640)	0.152
Proportional time for dressing changes	25	0.43 (0.35, 0.48)	25	0.42 (0.38, 0.47)	0.939

s* = seconds

All p-values were calculated using the Wilcoxon rank-sum test

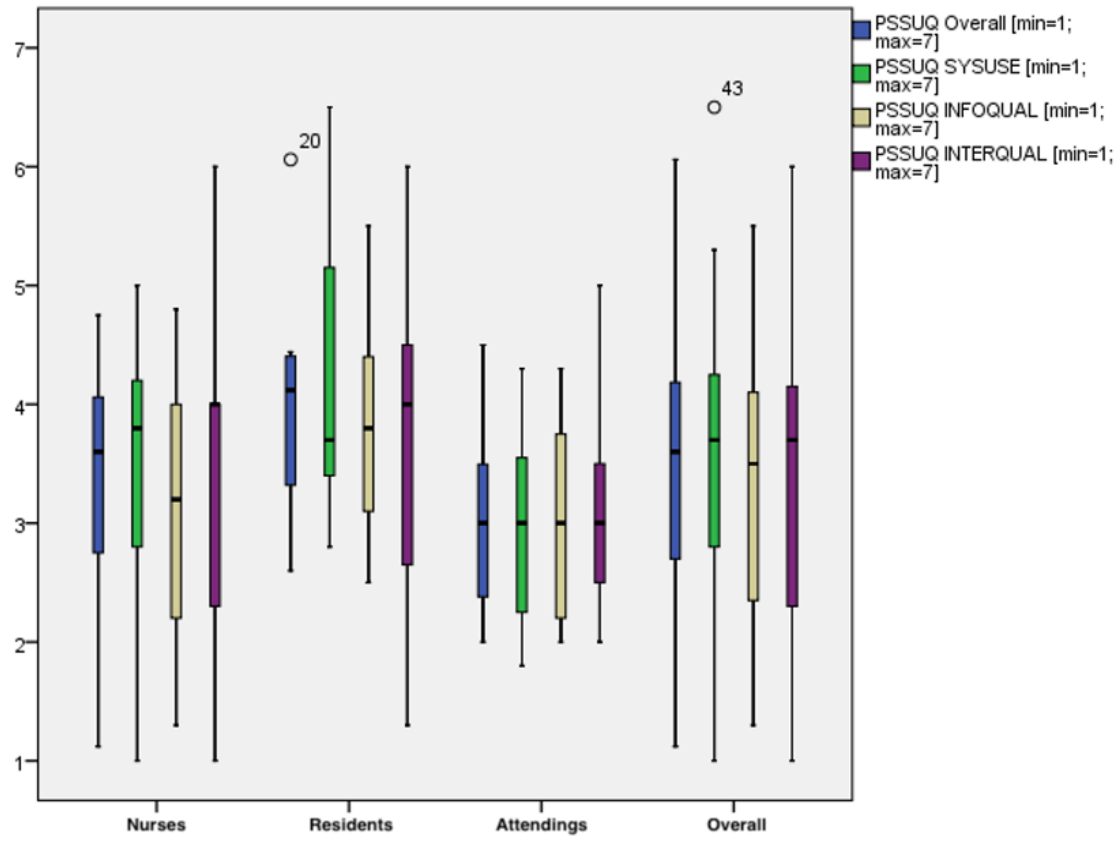

Figure S1: Detailed Chart View Across Professions for PSSUQ
